# EXAMINING THE IMPACT OF BURNOUT ON RESEARCH PARTICIPATION IN CLINICIANS TREATING DEMENTIA

**DOI:** 10.1093/geroni/igad104.3123

**Published:** 2023-12-21

**Authors:** Anam Rawoof, Emily Summerhayes, Sonia Talwar, Nancy Hodgson

**Affiliations:** University of Pennsylvania, Philadelphia, Pennsylvania, United States; University of Pennsylvania, Philadelphia, Pennsylvania, United States; University of Pennsylvania, Philadelphia, Pennsylvania, United States; University of Pennsylvania, Philadelphia, Pennsylvania, United States

## Abstract

Clinicians treating people living with dementia (PLWD) are likely to face burnout due to 1) the significant emotional burden of the job and 2) the increasing number of patients requiring dementia care. While much research has been conducted on the impact of clinician burnout on patient care and outcomes, more research is needed on its effects on clinician participation in research studies. This study will analyze qualitative data to examine the effect of clinician burnout on participation and intervention uptake in a dementia care research study.

